# Undesirable effect of cosmetic lip augmentation with autologous fat tissue

**DOI:** 10.1186/1756-0500-6-79

**Published:** 2013-03-05

**Authors:** Estela Kaminagakura, Jucely Aparecida Rosa, Yasmin Rodarte Carvalho, Janete Dias Almeida

**Affiliations:** 1 Discipline of Stomatology, Department of Biosciences and Oral Diagnosis, São José dos Campos Dental School, UNESP - Univ Estadual Paulista, Engenheiro Francisco José Longo, 777, São José dos Campos, SP, 12245-000, Brazil; 2 Postgraduate Program in Biopathology, Department of Biosciences and Oral Diagnosis, São José dos Campos Dental School, UNESP - Univ Estadual Paulista, José dos Campos, Brazil; 3 Discipline of Oral Pathology, Department of Biosciences and Oral Diagnosis, São José dos Campos Dental School, UNESP - Univ Estadual Paulista, José dos Campos, Brazil

**Keywords:** Cosmetic filler, Double lip, Dermal filler

## Abstract

**Background:**

Facial cosmetic procedures are commonplace nowadays, especially techniques that aim to increase lip volume. Full lips provide a youthful, healthy, feminine and sensual appearance. There are many techniques and materials used to recover the loss of contour that occurs with age.

**Case presentation:**

An unusual case of fat tissue accumulation following cosmetic upper lip augmentation in a 61 year-old female was reported. Surgical treatment was performed for esthetic concerns. Microscopically, the tissue removed was composed of muscle fibers and mature adipocytes.

**Conclusion:**

Undesirable effects of esthetic treatment can occur and the clinician should be familiar with such complications to diagnose and manage them.

## Background

Full and well-defined lips represent beauty, attractiveness and sensuality [1,2]. Gravity, maxillomandibular bone resorption, teeth and soft tissue loss, sun exposure and smoking contribute to signs of aging on the lip [2,3]. Several types of treatment are described to restore a youthful appearance, ranging from surgical methods to filler materials, such as collagen, hyaluronic acid, polymethylmethacrylate (PMMA), synthetic hydrogels, calcium hydroxyapatite, silicone, expanded polytetrafluoroethylene and autologous fat graft [3]. This report discusses an unusual case of fat tissue accumulation following cosmetic lip augmentation.

## Case report

A 61 year-old female was attended at our department complaining about an unesthetic “fold” on her upper lip when smiling and talking. During anamnesis, she reported having type 2 diabetes, hypertension and a history of cosmetic augmentation of the upper lip. Twelve years ago, she was submitted to an injection of autologous fat tissue in her upper lip; however, she did not know any details about the procedure.

**Figure 1:**
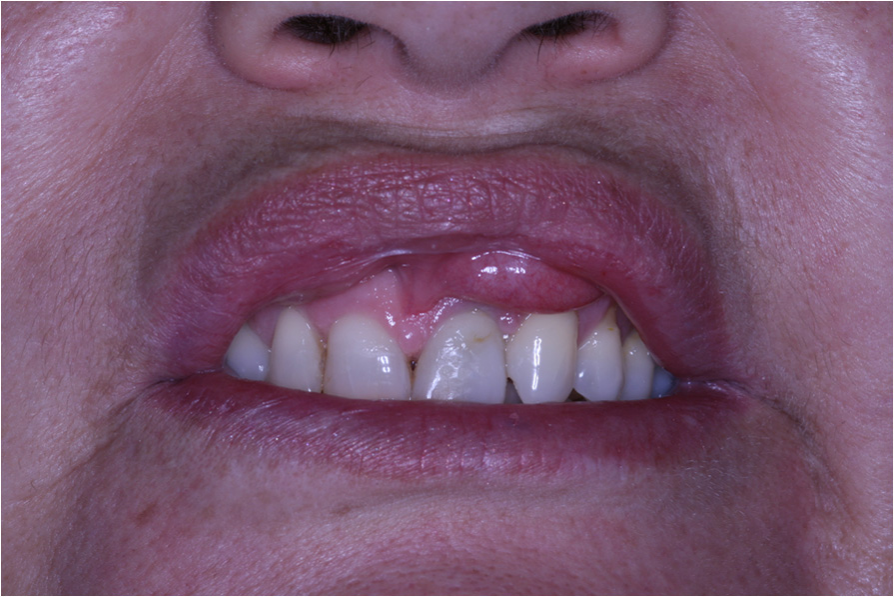
Initial clinical photograph of the enlargement on the upper lip.

**Figure 2:**
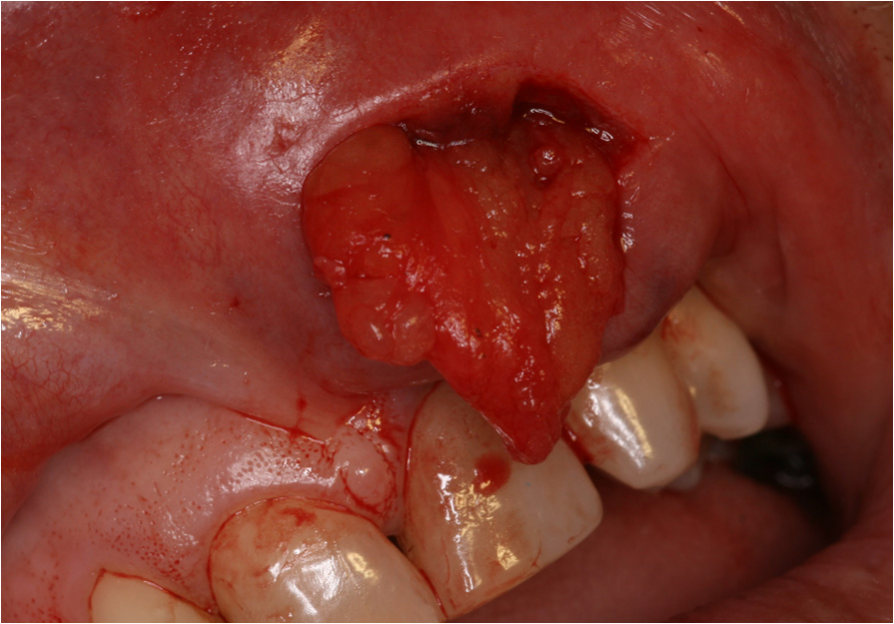
Photograph of surgery showing removal of the autologous fat graft.

**Figure 3:**
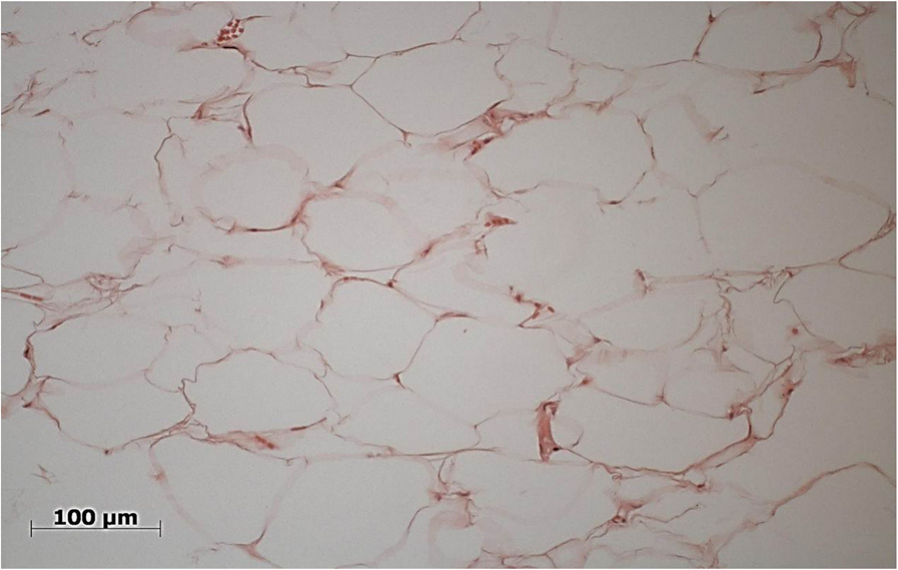
Photomicrography exhibiting mature adipocytes (HE staining).

**Figure 4:**
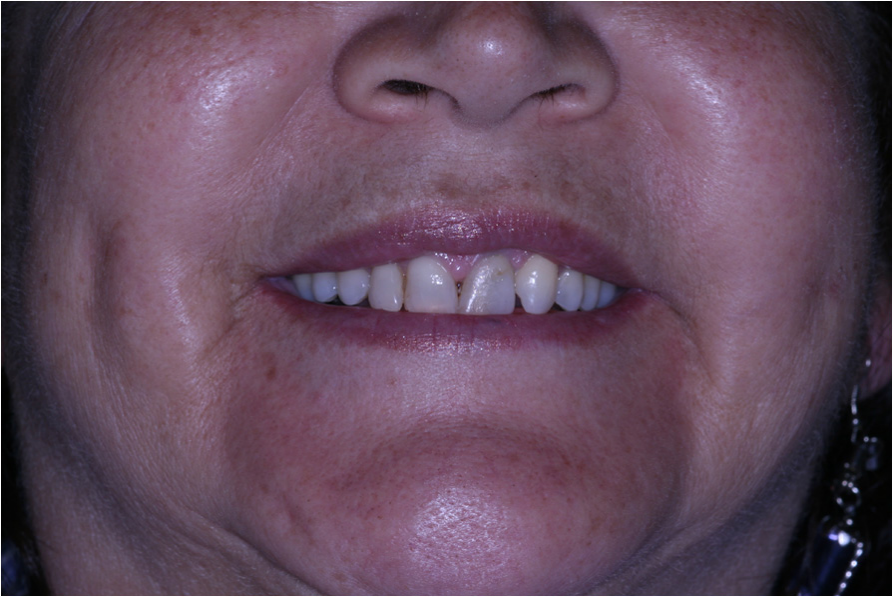
Final clinical appearance.

Physical examination revealed bilateral submucosal enlargement of the upper lip, which was soft on palpation, especially on the left side, and showed no color alteration of the mucosa (Figure 1). She was submitted to surgery based on the diagnostic hypotheses of dislocation of fat graft or double lip. The incision was made on the fold, revealing that the fat tissue was well demarcated, and was easily removed (Figure 2). Microscopically, the specimen was composed of muscle fibers and mature adipocytes (Figure 3). After 30 days of follow-up, she was satisfied with clinical result (Figure 4).

## Discussion

Autologous fat transplantation is indicated for the correction of wrinkles, depressed or atrophic areas in the face [1] and volume loss in the aging process [4]. Fat injections are more successful in facial areas due to the availability of a richer vascular supply [1]. Autologous fat implant is readily available, inexpensive, host compatible and can be harvested easily and repeatedly when needed, without promoting allergies or foreign body reactions [4]. However, the disadvantages of using this material are its high viscosity and lower rates of success, especially when used for changes that occur due to aging [1].

Longevity of the correction is unpredictable, depending on the harvesting and transfer techniques of the fat [5]. Undesired dislocation of the prosthetic material to more superficial parts of the lips could be attributed to frequent movement in this area [3,6] and ptosis induced by gravity. The lipofilling used in facial sites shows a very high risk of developing nodules of adipose tissue and other visible irregularities [4] that cause undesirable cosmetic effects, as occurred in this case. The amount of fat transferred can play a role in the degree of volume retention and in the onset of contour problems [7]. In addition, fat has an unpredictable resorption rate in the perioral region [2,3,7].

Differential diagnosis of this case was unsatisfactory esthetic treatment and double lip. Double lip is a rare oral abnormality, characterized by a deformity of the lip, in which a fold of labial mucosa is evident at rest or smiling [8]. Double lip, blepharochalasis and thyroid enlargement are features of Ascher Syndrome [9]. Microscopically, it presents as hyperplasia of the salivary gland and of squamous epithelium [8]. In this study, the patient only presented the appearance of “double lip”. Monhian et al. [10] have reported inflammatory response and fibrotic tissue without the presence of adipocytes in biopsies from local implantation of autologous fat graft. In this report, mature adipocytes and muscle fibers were observed. The final diagnosis was based on clinical exam and microscopic evidence.

Surgical treatment was performed for esthetic concerns. However, the treatment of patients with similar clinical appearance could be necessary due to alterations in phonetics, mastication and difficulty in wearing a prosthesis [8]. The patient decided not to perform surgery on the contralateral side and she was satisfied with the outcome.

## Conclusion

Nowadays, esthetic treatments are widely performed, especially in the perioral area. In some cases, undesirable effects can occur and the clinician should be familiar with such complications in order to diagnose and manage them correctly.

## Consent information

Written informed consent was obtained from the patient for publication of this case report and any accompanying images. A copy of the written consent is available for review by the Editor of this journal.

## Competing interests

The authors declare that there are no conflicts of interest.

## Authors’ contributions

EK, JAR and JDA were involved in clinical attendance and drafting the manuscript. YRC performed the microscopic analyse. All authors read and approved the final manuscript.
